# Development of a tool for predicting *HNF1B* mutations in children and young adults with congenital anomalies of the kidneys and urinary tract

**DOI:** 10.1007/s00467-023-06262-9

**Published:** 2024-01-10

**Authors:** Marcin Kołbuc, Mateusz F. Kołek, Rafał Motyka, Beata Bieniaś, Sandra Habbig, Kathrin Burgmaier, Larisa Prikhodina, Svetlana Papizh, Velibor Tasic, Christine Okorn, Maria Szczepańska, Katarzyna Kiliś-Pstrusińska, Anna Wasilewska, Piotr Adamczyk, Marcin Tkaczyk, Małgorzata Pańczyk-Tomaszewska, Monika Miklaszewska, Krzysztof Pawlaczyk, Ewelina Bukowska-Olech, Aleksander Jamsheer, Augustina Jankauskiene, Jens König, Hae Il Cheong, Yo Han Ahn, Sophie Kaspar, Przemysław Sikora, Bodo B. Beck, Marcin Zaniew

**Affiliations:** 1 Department of Pediatrics, University of Zielona Góra, Zielona Góra, Poland; 2 Medical University of Vienna, Vienna, Austria; 3 Department of Pediatric Nephrology, Medical University of Lublin, Lublin, Poland; 4 Department of Pediatrics, Faculty of Medicine and University Hospital Cologne, University of Cologne, Cologne, Germany; 5 Faculty of Applied Healthcare Science, Deggendorf Institute of Technology, Deggendorf, Germany; 6 Division of Inherited & Acquired Kidney Diseases, Veltishev Research Clinical Institute for Pediatrics & Children Surgery, Pirogov Russian National Research Medical University, Moscow, Russia; 7 Medical School Skopje, University Children’s Hospital, 1000 Skopje, North Macedonia; 8 Department of Pediatric Nephrology, University Hospital Essen, Essen, Germany; 9 Department of Pediatrics, Faculty of Medical Sciences in Zabrze, Medical University of Silesia, Katowice, Poland; 10 Department of Pediatric Nephrology, Wrocław Medical University, Wrocław, Poland; 11 Department of Pediatric Nephrology, University Hospital, Białystok, Poland; 12 Department of Pediatrics, Faculty of Medical Sciences in Katowice, Medical University of Silesia, Katowice, Poland; 13 Department of Pediatrics, Immunology and Nephrology, Polish Mother’s Memorial Hospital Research Institute, Łódź, Poland; 14 Department of Pediatrics and Nephrology, Medical University of Warsaw, Warsaw, Poland; 15 Department of Pediatric Nephrology and Hypertension, Jagiellonian University Medical College, Kraków, Poland; 16 Department of Nephrology, Transplantology and Internal Medicine, Poznan University of Medical Sciences, Poznań, Poland; 17 Department of Medical Genetics, Poznan University of Medical Sciences, Poznań, Poland; 18 Centers for Medical Genetics GENESIS, Poznań, Poland; 19 Pediatric Center, Institute of Clinical Medicine, Vilnius University, Vilnius, Lithuania; 20 Department of General Pediatrics, University Children’s Hospital Münster, Münster, Germany; 21 Department of Pediatrics, Seoul Red Cross Hospital, Seoul, South Korea; 22 Department of Pediatrics, Seoul National University College of Medicine, Seoul, South Korea; 23 Institute of Human Genetics and Center for Molecular Medicine Cologne, University of Cologne, Faculty of Medicine and University Hospital Cologne, Cologne, Germany

**Keywords:** CAKUT, *HNF1B*, Hypomagnesemia, FEMg

## Abstract

**Background:**

We aimed to develop a tool for predicting *HNF1B* mutations in children with congenital abnormalities of the kidneys and urinary tract (CAKUT).

**Methods:**

The clinical and laboratory data from 234 children and young adults with known *HNF1B* mutation status were collected and analyzed retrospectively. All subjects were randomly divided into a training (70%) and a validation set (30%). A random forest model was constructed to predict *HNF1B* mutations. The recursive feature elimination algorithm was used for feature selection for the model, and receiver operating characteristic curve statistics was used to verify its predictive effect.

**Results:**

A total of 213 patients were analyzed, including *HNF1B*-positive (mut + , *n* = 109) and *HNF1B*-negative (mut − , *n* = 104) subjects. The majority of patients had mild chronic kidney disease. Kidney phenotype was similar between groups, but bilateral kidney anomalies were more frequent in the mut + group. Hypomagnesemia and hypermagnesuria were the most common abnormalities in mut + patients and were highly selective of *HNF1B*. Hypomagnesemia based on age-appropriate norms had a better discriminatory value than the age-independent cutoff of 0.7 mmol/l. Pancreatic anomalies were almost exclusively found in mut + patients. No subjects had hypokalemia; the mean serum potassium level was lower in the *HNF1B* cohort. The abovementioned, discriminative parameters were selected for the model, which showed a good performance (area under the curve: 0.85; sensitivity of 93.67%, specificity of 73.57%). A corresponding calculator was developed for use and validation.

**Conclusions:**

This study developed a simple tool for predicting *HNF1B* mutations in children and young adults with CAKUT.

**Graphical abstract:**

**Figure:**
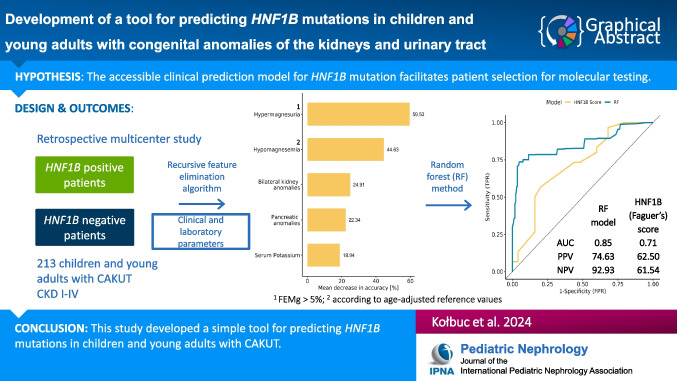
A higher resolution version of the Graphical abstract is available as Supplementary information

**Supplementary Information:**

The online version contains supplementary material available at 10.1007/s00467-023-06262-9.

## Introduction

Mutations in the *HNF1B* gene are the leading monogenic cause of congenital anomalies of the kidneys and urinary tract (CAKUT) [1–4], which, in turn, is a common cause of kidney failure [5]. The product of this gene, i.e., hepatocyte nuclear factor 1β (HNF1β), is expressed in many organs, predominantly the kidney, liver, intestine, pancreas, and urogenital tract. Patients with *HNF1B*-related disease may exhibit organ involvement and a wide range of biochemical disturbances. Thus, the spectrum of phenotypes in patients harboring pathogenic *HNF1B* variants is pleotropic [6]. The kidney phenotype is highly variable, yet it is the earliest and most frequent manifestation [6–8]. The involvement of other organs makes patients very vulnerable. Given this, patients with *HNF1B*-associated disease may have special medical needs and require multispecialized care. In this respect, the molecular diagnosis of *HNF1B*-related disease is crucial, as early diagnosis allows for personalized care. Accurate patient selection is also essential in terms of a cost-effective approach, which is of special importance in areas with limited resources. However, selecting appropriate patients for genetic testing remains a challenge for clinicians [9, 10].

The benefits of timely detection and the challenges involved in making the diagnosis of *HNF1B* disease have driven clinicians to develop tools to predict the likelihood of an *HNF1B* mutation in patients with CAKUT. Faguer et al. [9] proposed a scoring system based on 17 parameters, with kidney hyperechogenicity and/or cysts and genital and pancreatic abnormalities scoring highest, followed by other features, such as abnormal fetal kidney ultrasound, positive family history, kidney hypoplasia or dysplasia, and hypomagnesemia. However, perhaps due to its complexity, this scoring system is not widely used in clinical practice [6] and requires revision in the pediatric population [11]. One of the most important limitations of the score is that it was established in patients of all ages, which calls into question its use in children. To improve the score, Raaijmakers et al. [12] proposed to modify Faguer’s criteria, which were validated in a prospective manner. However, this approach yielded a low detection rate (10%). Therefore, the authors, based on a post hoc analysis of their results, suggested consideration of *HNF1B* mutations in patients with bilateral kidney anomalies, with kidney dysplasia and cysts, as well as concomitant hypomagnesemia. Importantly, the above criteria proposed by Faguer and Raaijmakers were developed in a mixed population of adults and children by using adult standards to define disease-specific disorders, such as hypomagnesemia and hyperuricemia. In fact, the reference values for serum magnesium (sMg) and serum uric acid (sUA) are highly dependent on age [13–15]. Considering the age-dependent variability in biochemical parameters and the dynamic nature of the disease, pediatric patients, for whom a uniform definition of biochemical disorders should not be applied, warrant a special approach. In fact, Kołbuc et al. [16] showed that hypomagnesemia is underdiagnosed in children with *HNF1B* due to applying an inaccurate lower limit of normal for sMg.

Thus, this study aimed to establish an accurate and simple predictive model based on clinical and laboratory data and to design a corresponding calculator of *HNF1B* probability for clinical use. Our goal was to develop a tool dedicated to children with CAKUT. Hence, we utilized the most recent age-appropriate norms to define biochemical abnormalities.

## Materials and methods

### Study population

**Fig. 1:**
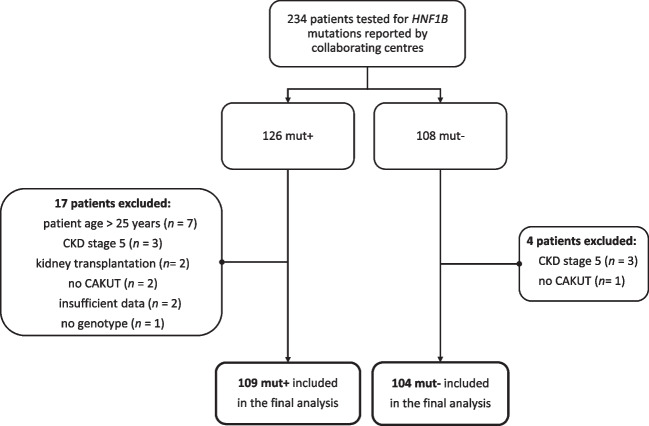
Flowchart of patient selection

This retrospective multicenter study was designed to collect data from children and young adults with known *HNF1B* mutation status. The main source of the study cohort was the Polish Registry of Inherited Tubulopathy (POLtube), which oversees the recruitment and data collection of tubular disorders and other rare kidney diseases including *HNF1B* nephropathy in Poland. Additionally, to increase the number of individuals to make statistical analysis possible, and to eliminate a possible selection bias, we identified study subjects by approaching physicians and research groups from other countries, who had collaborated with us in previous studies. A spreadsheet with requested information was sent to clinicians with a request to provide clinical, biochemical, and genetic results of patients with known *HNF1B* status. Patient selection for molecular testing was at clinicians’ discretion and was not subject to analysis in our study. Informed consent for the study was obtained individually according to local regulations in each participating center/country. Ethical approval was waived by the local Ethics Committee of the University of Zielona Gora in light of the retrospective nature of this study. The exclusion criteria included age > 25 years, chronic kidney disease stage 5 or post-kidney transplant status, absence of anomaly of kidney and urinary tract, insufficient clinical data, or lack of genotype. Between January 2021 and October 2022, data from 234 patients were collected. Of these, the records of 21 subjects were discarded (Fig. 1). The study cohort ultimately comprised 213 patients, of whom 109 subjects were positive for *HNF1B* mutations (mut +) and 104 were negative (mut −). The patients were from Poland (66.7%), Germany (12.7%), South Korea (12.7%), Russia (4.7%), North Macedonia (1.7%), and Lithuania (1.5%).

### Clinical assessment and definitions of analyzed parameters

Anonymized data provided by referring physicians were retrospectively reviewed. Data from the time of genetic testing were used, and if unavailable, from the latest visit. The CAKUT phenotype was defined according to the sonographic criteria suggested by Faguer et al. [9]. Patients were also classified as having a major or minor kidney anomaly, according to Raaijmakers et al. [12].

Hypomagnesemia and hyperuricemia were defined according to age and sex (for sUA) reference values [15]. To demonstrate the effect of an appropriate cutoff value for the definition of hypomagnesemia, we used a conservative lower limit for sMg of 0.7 mmol/l. Hypokalemia was defined as a serum potassium level < 3.5 mmol/l, and hyperparathyroidism was defined as a parathyroid hormone (PTH) level > 65 pg/ml. Patients receiving supplements of magnesium or potassium or those being treated with allopurinol were also considered to have hypomagnesemia, hypokalemia, or hyperuricemia, respectively. The raw sMg, serum potassium, and sUA values of these patients and those who were receiving diuretics or renin–angiotensin–aldosterone system inhibitors were not included in the analysis. Hypermagnesuria was defined as a fractional excretion of magnesium (FEMg) > 5% [16–18], and hypocalciuria as a fractional excretion of calcium < 1%. The estimated glomerular filtration rate (eGFR) was calculated using the Schwartz equation for patients < 20 years of age [19, 20] or the Chronic Kidney Disease Epidemiology Collaboration (CKD-EPI) equation for those who were > 20 years of age [21]. The subject’s severity of chronic kidney disease (CKD) was classified according to the National Kidney Foundation Kidney Disease Outcomes Quality Initiative CKD staging system, considering physiologically lower GFR in children < 2 years [22]. Standard deviation scores (SDS) for height and body mass index (BMI) were calculated based on World Health Organization growth charts (http://www.who.int/growthref/en). Growth impairment was defined as height-SDS <  − 2, overweight as BMI-SDS > 1, or obesity when BMI-SDS > 2.

Genetic testing was conducted locally, and molecular results were reported by the referring physician. In the vast majority of patients (79.8%), mutational analysis was performed by targeted Sanger sequencing of exons 1–9 of the *HNF1B* gene, and by MLPA analysis to detect copy number variations. In a small proportion of patients, CAKUT/PKD multigene panels were performed (16.9%) or whole-exome sequencing (3.3%). All variants were evaluated using online databases, including HGMD Professional, OMIM, PubMed, ClinVar, and VarSome databases. The pathogenicity of the variants was classified using the American College of Medical Genetics and Genomics criteria.

### Statistical analysis

Data are presented as means (95% confidence interval) for continuous variables and as counts (percentages) for categorical variables. Hypotheses of differences between mut + and mut − groups were tested using Welch’s independent-samples *t* test for continuous variables and Fisher’s exact test for categorical variables. Cohen’s *d* and odds ratios (*OR*) were used as effect size coefficients. Pearson’s correlation coefficient was used to investigate the strength of correlations.

The subjects were randomly divided into a training set (70%) and a test (validation) set (30%). To choose the best algorithm for the construction of a predictive model for the *HNF1B* mutation, various machine learning algorithms were tested. The recursive feature elimination (RFE) algorithm was used for feature selection into the model, and receiver operating characteristic (ROC) curve statistics were used to verify its predictive effect. As a global statistical significance level, *α* = 0.050 was used. All analyses were performed using R, SQL, and Python programming languages supported by multiple packages.

## Results

### Patient characteristics

The clinical characteristics of the two cohorts (mut + and mut −) are highlighted in Table 1. Both groups were comparable with respect to age, gender distribution, and anthropometric parameters. The majority of patients had CKD stage 1 or stage 2 (88.24% and 83.67% of mut − and mut + , respectively). The mean eGFR was slightly lower in the mut + group compared to the mut − group (*p* = 0.025). However, impaired kidney function was not suggestive of the *HNF1B* mutation.

**Table 1:** Characteristics of the study groups

		Presence of *HNF1B* mutation			
	*n*	Mut − (*n* = 104)^a^	Mut + (*n* = 109)^a^	Effect size^b^	*p* value^c^
Age (at molecular testing)	213	9.39 (8.23–10.56)	8.98 (7.85–10.11)	0.07	0.619
Female		40 (38.46%)	41 (37.61%)		
Male		64 (61.54%)	68 (62.39%)		
Height-SDS	186	− 0.03 (− 0.36 to 0.29)	− 0.05 (− 0.33 to 0.23)	0.01	0.950
Short stature	186	7 (8.54%)	9 (8.65%)	1.02	> 0.999
BMI-SDS	183	0.17 (− 0.12 to 0.46)	0.09 (− 0.20 to 0.37)	0.06	0.696
Overweight/obesity	183	21 (25.61%)	29 (28.71%)	1.17	0.739
eGFR (mL/min/1.73 m^2^)	181	110.63 (101.39–119.86)	97.05 (89.71–104.40)	0.34	0.025
CKD 1	181	57 (67.06%)	56 (57.14%)	0.58	0.092
CKD 2	181	18 (21.18%)	26 (26.53%)	1.30	0.490
CKD 3	181	9 (10.59%)	14 (14.29%)	1.37	0.512
CKD 4	181	1 (1.18%)	2 (2.04%)	1.71	> 0.999
CKD 5	181	0 (0.00%)	0 (0.00%)		
sMg (mmol/L)	170	0.86 (0.84–0.88)	0.73 (0.71–0.76)	1.17	< 0.001
Hypomagnesemia^d^	187	5 (5.75%)	59 (59.00%)	23.60	< 0.001
Hypomagnesemia (sMg < 0.7 mmol)	187	5 (5.75%)	38 (38.00%)	10.05	< 0.001
Serum potassium (mmol/L)	164	4.60 (4.50–4.70)	4.28 (4.21–4.36)	0.78	< 0.001
Hypokalemia	163	0 (0.00%)	0 (0.00%)		
sUA (mg/dl)	199	5.04 (4.71–5.38)	5.46 (5.15–5.77)	− 0.26	0.073
Hyperuricemia^d^	209	21 (20.39%)	43 (40.57%)	2.67	0.002
PTH (pg/ml)	115	43.44 (34.91–51.97)	57.17 (47.56–66.78)	− 0.39	0.038
Hyperparathyroidism	122	10 (19.61%)	25 (35.21%)	2.23	0.070
FEMg (%)	129	3.99 (3.41–4.57)	7.17 (6.17–8.17)	− 0.98	< 0.001
Hypermagnesuria (FEMg > 5%)	129	15 (20.83%)	37 (64.91%)	7.03	< 0.001
FEUA (%)	131	6.66 (5.98–7.33)	7.53 (6.00–9.05)	− 0.18	0.310
FECa (%)	143	0.49 (0.37–0.61)	0.49 (0.27–0.70)	0.01	0.953
FEK (%)	84	7.59 (5.56–9.61)	11.34 (8.80–13.88)	− 0.49	0.026
Diabetes mellitus	194	3 (2.88%)	10 (11.11%)	4.21	0.040
Pancreatic anomalies^e^	213	1 (0.96%)	13 (11.93%)	13.95	0.001
Liver involvement^e^	213	3 (2.88%)	14 (12.84%)	4.96	0.010
Developmental delay	213	3 (2.88%)	11 (10.09%)	3.78	0.050
Genital tract anomalies^e^	213	5 (4.81%)	9 (8.26%)	1.78	0.410
Positive family history^f^	213	36 (34.62%)	46 (42.20%)	1.38	0.264
Faguer score (points)	213	8.61 (7.51–9.70)	12.79 (11.62–13.96)	− 0.70	< 0.001

*BMI-SDS* body mass index standard deviation score, *CKD* chronic kidney disease, *eGFR* estimated glomerular filtration rate, *FECa* fractional excretion of calcium, *FEK* fractional excretion of potassium, *FEMg* fractional excretion of magnesium, *FEUA* fractional excretion of uric acid, *Height-SDS* height standard deviation score, *mut* + *HNF1B* positive patients, *mut − HNF1B* negative patients, *PTH* parathyroid hormone, *sMg* serum magnesium, *sUA* serum uric acid

^a^Mean (95% confidence interval) for continuous; n (%) for categorical

^b^Cohen’s *d* for continuous, odds ratio for categorical

^c^Welch two-sample *t* test for continuous, Fisher’s exact test for categorical

^d^According to reference values suggested by Ridefelt et al.[15]

^e^Described in detail in Suppl. Table S1

^f^Family member with either diabetes mellitus and/or structural kidney disease

Not surprisingly, we found hypermagnesuria (64.90%), hypomagnesemia (59.00%), hyperuricemia (40.57%), liver involvement (12.84%), and diabetes mellitus (DM; 11.11%) to be more common in mut + than in mut − subjects. Pancreatic anomalies were proven in 11.93% of patients and were almost exclusively found in those with *HNF1B* mutations (*OR* 13.95, *p* = 0.001). Organ involvement is presented in more detail in Supplementary Table S1. None of the subjects presented with hypokalemia; however, the mean serum potassium level was lower, and the fractional excretion of potassium was higher in the mut + group than in the mut − group (*p* < 0.001 and *p* = 0.026, respectively).

The kidney phenotype is presented separately for the two cohorts (Table 2). Cystic kidney disease and multicystic dysplastic kidney were the most common phenotypes in both cohorts. Their frequencies, similar to the other kidney phenotypes, were similar between groups. Bilateral anomalies were observed more frequently in the mut + group and had a high discriminative value (*OR* 4.85, *p* < 0.001). Notably, the percentage of major and minor kidney anomalies did not differ between groups.

**Table 2:** Kidney phenotype

		Presence of *HNF1B* mutation			
	*n*	Mut − (*n* = 104)^a^	Mut + (*n* = 109)^a^	Effect size^b^	*p* value^c^
Bilateral anomalies	213	65 (62.50%)	97 (88.99%)	4.85	< 0.001
Unilateral anomalies	213	39 (37.50%)	11 (10.09%)	0.19	< 0.001
Kidney cysts	213	58 (55.77%)	63 (57.80%)	1.09	0.783
Multicystic dysplastic kidney	213	19 (18.27%)	30 (27.52%)	1.70	0.142
Kidney hypodysplasia	213	12 (11.54%)	16 (14.68%)	1.32	0.547
Solitary kidney	213	14 (13.46%)	6 (5.50%)	0.37	0.060
Urinary tract malformations	213	11 (10.58%)	21 (19.27%)	2.02	0.086
Major anomalies^d^	213	96 (92.31%)	104 (95.41%)	1.73	0.400
Minor anomalies^d^	213	8 (7.69%)	6 (5.50%)	0.70	0.588

^a^Mean (95% confidence interval) for continuous; *n* (%) for categorical

^b^odds ratio

^c^Fisher’s exact test

^d^According to classification suggested by Raaijmakers et al.[12]

### Genetic results

Detailed information on the status of the detected variants is presented in Supplementary Table S2. Whole gene deletion was the most common mutation (55.04%) (Supplementary Table S3). One hundred and four patients (95.4%) had a pathogenic or likely pathogenic variant including five alterations classified as variants of uncertain significance (VUS). The clinical phenotype of patients carrying VUS is shown in Supplementary Table S4. Nine variants were novel. In patients who had multigene panels or whole-exome sequencing, no other CAKUT-related genes were found.

When comparing the two mutational groups, we found lower eGFR (81.73 mL/min/1.73 m^2^; 95% CI: 71.39–92.08) in individuals with point mutations compared to those with gene deletions (110.60 mL/min/1.73 m^2^; 95% CI: 101.63–119.57, *p* < 0.001). Although there were statistically significant differences in the mean concentrations of sMg (0.7 mmol/l; 95% CI: 0.67–0.73 vs. 0.77 mmol/l; 95% CI: 0.73–0.81, *p* = 0.017, in mut + and mut − , respectively), the frequency of hypomagnesemia did not differ between the two groups. Pancreas anomalies were more common in individuals with point mutations (*p* = 0.035), while developmental delay was reported only in those with whole gene deletions (*p* < 0.001).

### Assessment of magnesium homeostasis

Hypomagnesemia and hypermagnesuria were the most common abnormalities in mut + patients. Low sMg levels were tenfold more frequent in the mut + group when age-appropriate reference values were applied (59.00% vs. 5.75%). Applying the conservative cutoff for sMg (0.7 mmol/l) reduced the frequency of hypomagnesemia in the mut + cohort by more than 20%. Likewise, hypomagnesemia, according to age-dependent norms, had a better discriminatory value (*OR* 23.60 vs. 10.05, respectively). There was no change in the mut − group in this regard.

**Fig. 2:**
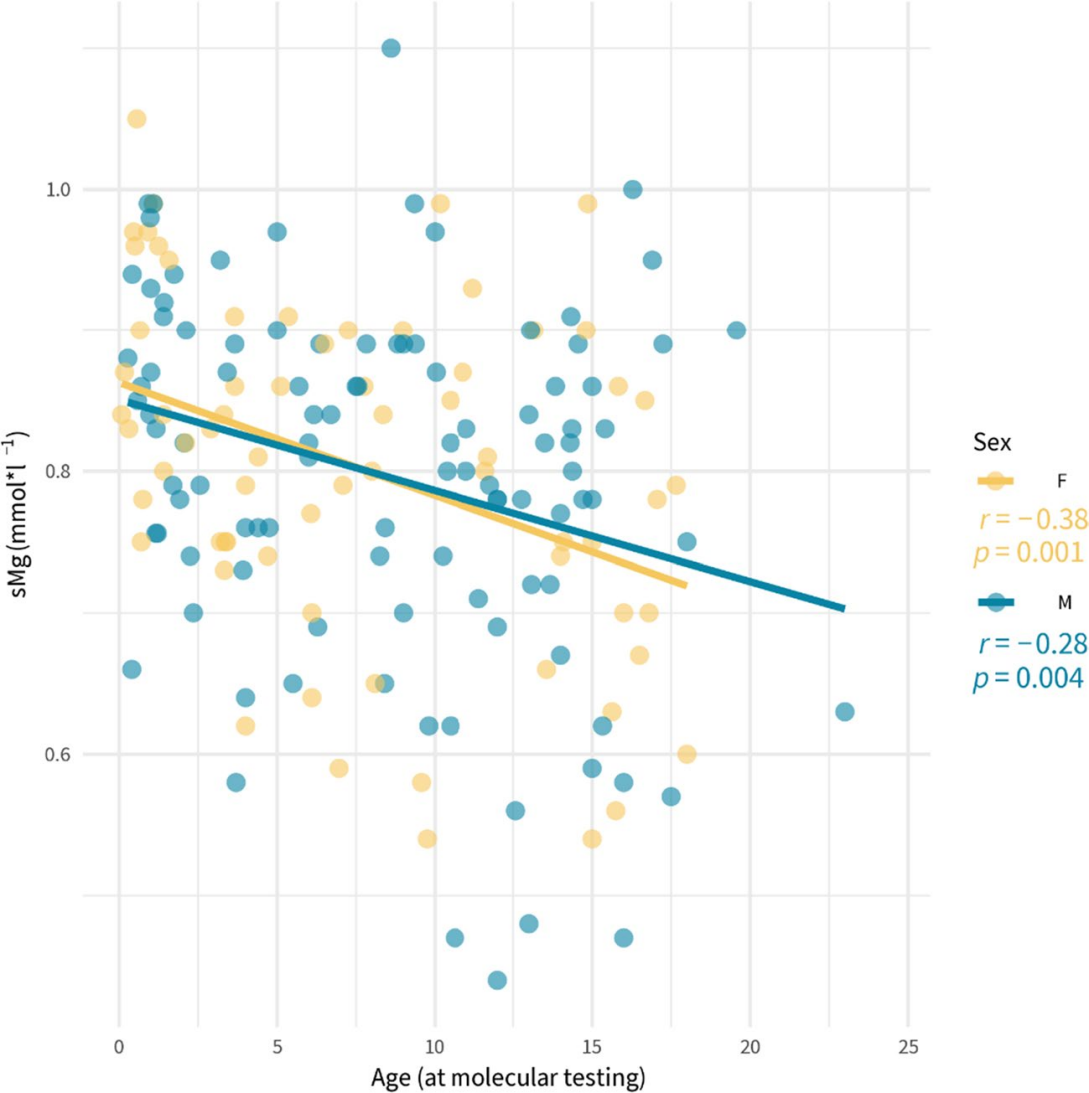
Correlations between serum magnesium concentrations and age shown separately for males (M) and females (F)

As expected, the correlation analysis in the entire cohort showed the effect of age on sMg (*r* =  − 0.38, *p* = 0.001 for females; *r* =  − 0.28, *p* = 0.004 for males) (Fig. 2). Thus, hypomagnesemia was also analyzed in two age subgroups (i.e., 0.5–3- and 4–17-year intervals) [14]. The frequency of hypomagnesemia remained higher in mut + in the analyzed age intervals (Table 3). At the age of 0.5–3 years, hypomagnesemia was exclusively found in the mut + group. Importantly, when using the age-adjusted reference values, it was observed in 42.31% of mut + patients, while when using the cutoff of 0.7 mmol/l, hypomagnesemia was 11-fold lower (3.85%).

**Table 3:** Frequency of hypomagnesemia according to age intervals

Age intervals	Hypomagnesemia mut + (*n*, %)		Hypomagnesemia mut − (*n*, %)		*p* value^a^	*p* value^b^
	Age-dependent norms	sMg < 0.7 mmol/l	Age-dependent norms	sMg < 0.7 mmol/l		
0.5–3 yeas	11/26 (42.31%)	1/26 (3.85%)	0/17 (0.00%)	0/17 (0.00%)	< 0.001	> 0.999
4–17 years	46/68 (67.65%)	35/68 (51.47%)	5/62 (8.07%)	5/62 (8.07%)	< 0.001	< 0.001

*sMg* serum Mg level

^a^Comparison between the groups when hypomagnesemia defined by age-dependent norms [15]

^b^Comparison between the groups when hypomagnesemia defined by sMg < 0.7 mmol/l

A similar effect of applying an appropriate limit of sMg in the older age interval (4–17 years) was observed, but the difference was not that significant (67.65% vs. 51.47%). The abovementioned differences were not noticeable in the mut − group, regardless of the cutoff applied.

### Construction of a random forest–based predictive model

All subjects’ data were randomly divided into a training (70%) and a test set (30%). Columns with more than 50% missing data (i.e., fractional excretion of potassium) were excluded from further analysis to ensure sufficient quality of imputation. Missing values in the data were imputed using the Multiple Imputation by Chained Equation (MICE) algorithm for each dataset separately. The imputation of missing data resulted in 24 different training datasets. To avoid overfitting, they were not combined into one large dataset but were analyzed independently, and the results were then pooled.

To choose the best algorithm for the construction of a predictive model, various machine learning algorithms were tested (Supplementary Figure S1). The results for all 24 datasets were pooled according to Rubin’s rules to obtain an overall summary of algorithm performance. The algorithm with the highest accuracy was random forest (RF), which was used for further analysis and building of the predictive model.

**Fig. 3:**
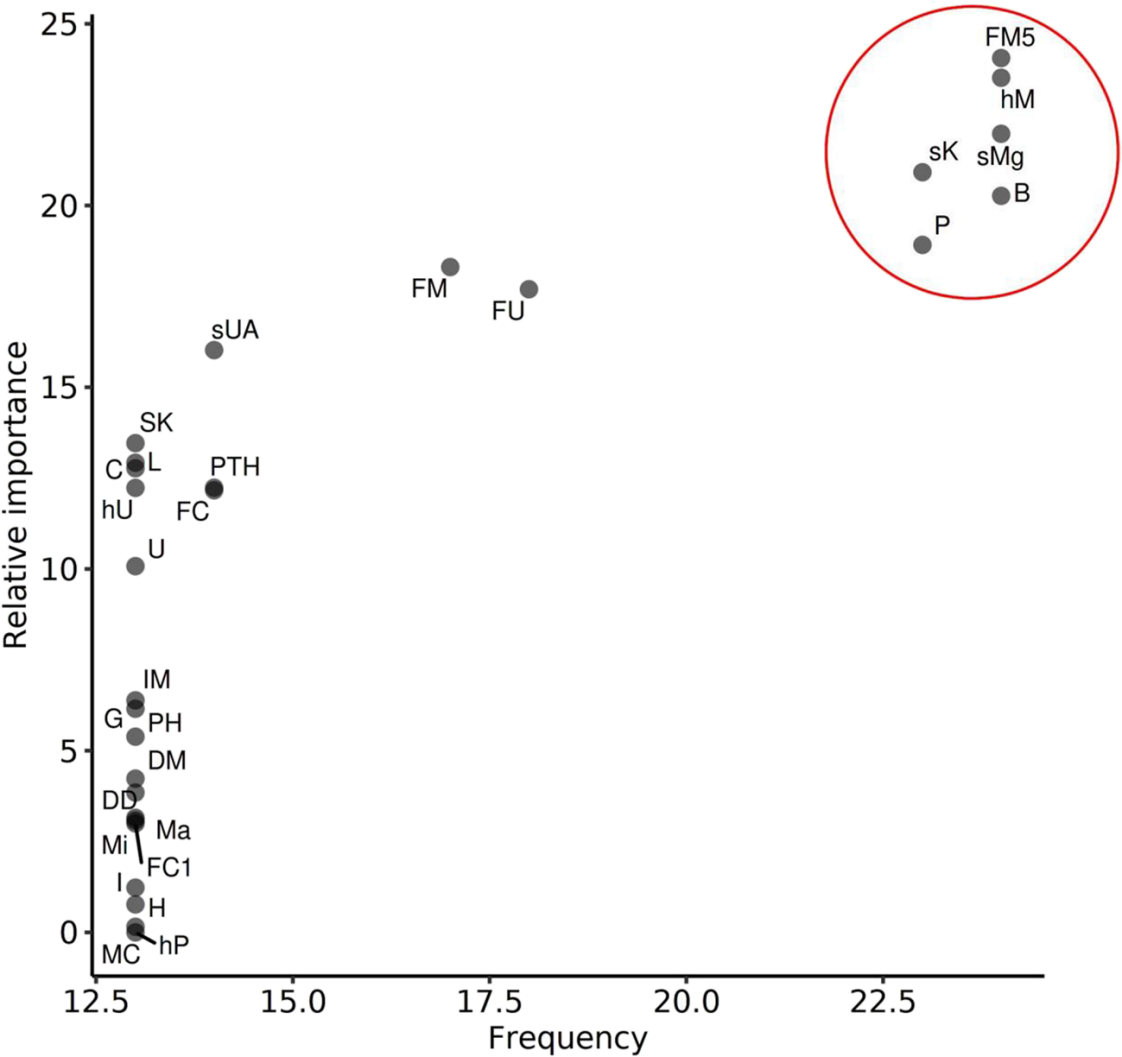
Scatterplot of relative importance and frequency of occurrences for each variable derived from the recursive feature elimination algorithm. In the top right-hand corner, a well-differentiated cluster of relevant variables is depicted. B, bilateral kidney affection; C, kidney cysts; DD, developmental delay; DM, diabetes mellitus; FC, fractional excretion of calcium value; FC1, hypocalciuria (FECa < 1%); FM, fractional excretion of Mg value; FM5, hypermagnesuria (FEMg > 5%); FU, fractional excretion of uric acid; G, genital tract anomaly; hP, hyperparathyroidism; hU, hyperuricemia; hM, hypomagnesemia; H, kidney hypodysplasia; I, impaired fasting glucose; IM, hyperglycemia (IFG and/or DM); L, liver involvement; Ma, major kidney anomaly; MC, multicystic kidney disease; Mi, minor kidney anomaly; P, pancreas anomaly; PH, positive history; PTH, parathyroid hormone; sK, serum potassium; sMg, serum magnesium; SK, solitary kidney; sUA, serum uric acid; U, urinary tract malformation

Feature selection for the model was performed using the RFE algorithm. For every variable used for modeling, the number of occurrences in the 24 datasets and the relative variable importance in each dataset were assessed to perform subsequent feature selection. We assumed that variables with a higher number of occurrences and higher relative importance were more likely to accurately predict the occurrence of *HNF1B* mutation. When plotting these two variables on a scatterplot, a clear pattern emerged, which allowed us to choose the possible predictors (Fig. 3). As the variables sMg and hypomagnesemia tended to explain the same portion of the variance in the model, two separate models were tested, and the one with better accuracy was chosen (data not shown). The final variables selected for the modeling were FEMg, hypomagnesemia (better accuracy in the model than sMg level), bilateral kidney anomalies, pancreatic malformations, and serum potassium. Supplementary Figure S2 shows the importance of each variable.

**Fig. 4:**
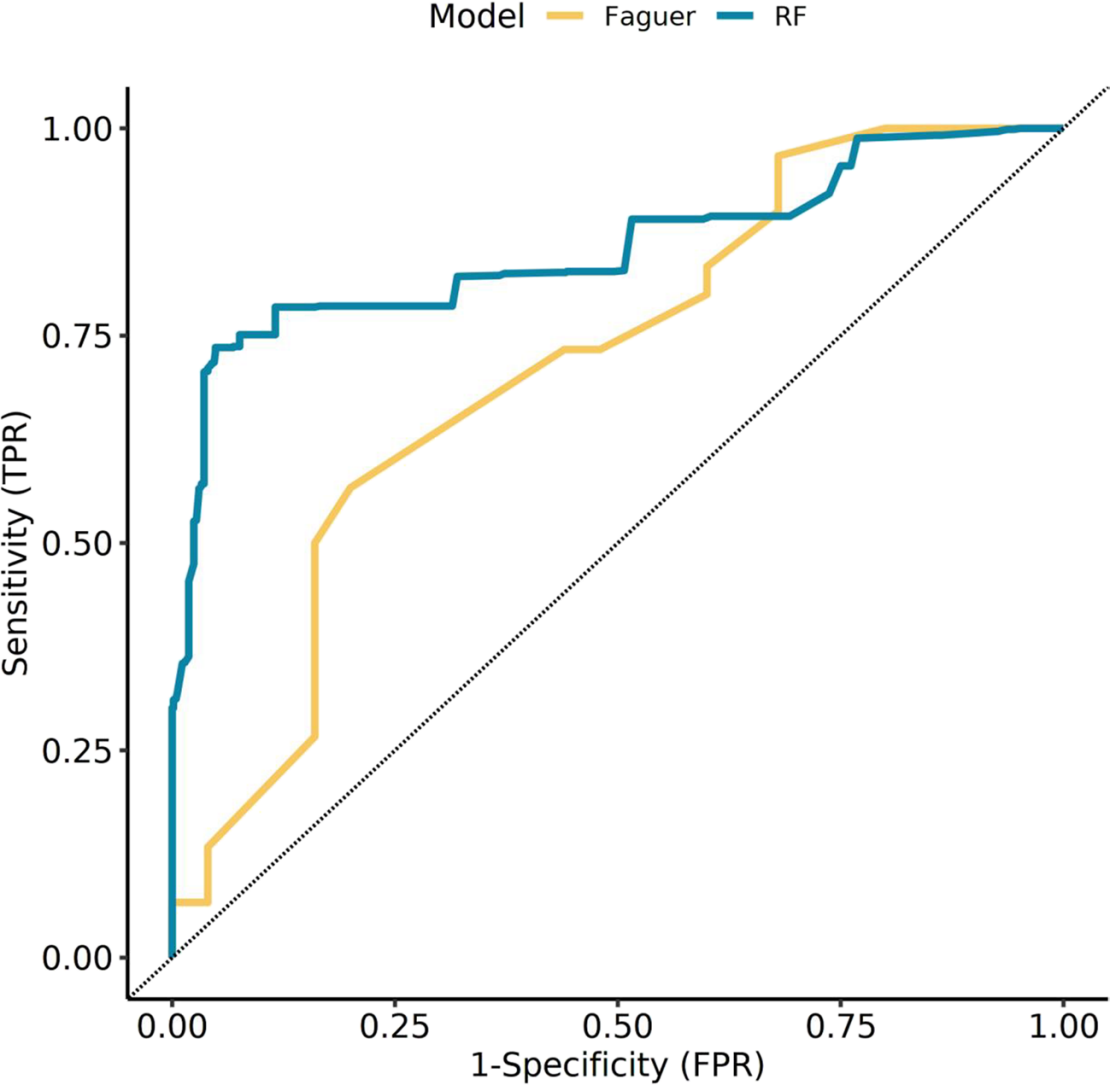
The receiver operating characteristic curves for the random forest (RF) based model and the *HNF1B* score (Faguer’s score) on the test dataset. Area under the curve for the RF model was 0.85 (95% CI; 0.84–0.87) versus 0.71 (95% CI; 0.69–0.74) for Faguer’s score

We conducted hyperparameter tuning and fitting of the final model with a predefined formula. The model was fitted to each imputed dataset separately, and then the votes of all subsequent models were counted and summarized. Then, predictions on the training and test datasets were made. The final model performance is presented in Table 4. The ROC curve on the validation dataset is shown in Fig. 4. The area under the curve (AUC) of our model was 0.85, with a sensitivity of 93.67% and a specificity of 73.57%. For comparison, the performance results of the *HNF1B* (Faguer’s) score are also shown.

**Table 4:** Comparison of random forest-based model performance with the *HNF1B* (Faquer’s) score on the training and test datasets

	Random forest model		*HNF1B* score	
	Training	Test	Training	Test
Accuracy (%)	88.67 (82.48–93.26)	82.53 (80.54–84.40)	63.33 (55.08–71.04)	61.82 (59.34–64.25)
AUC	0.89 (0.84–0.94)	0.85 (0.84–0.87)	0.73 (0.65–0.81)	0.71 (0.69–0.74)
Sensitivity (%)	91.78 (82.96–96.92)	93.29 (91.17–95.03)	43.84 (32.24–55.95)	40.00 (36.35–43.74)
Specificity (%)	85.71 (75.87–92.65)	73.57 (70.45–76.53)	81.82 (71.38–89.69)	80.00 (77.13–82.66)
PPV (%)	85.90 (76.17–92.74)	74.63 (71.61–77.48)	69.57 (54.25–82.26)	62.50 (57.83–67.00)
NPV (%)	91.67 (82.74–96.88)	92.93 (90.71–94.76)	60.58 (50.51–70.02)	61.54 (58.58–64.44)
Prevalence (%)	48.67 (40.43–56.95)	45.45 (42.95–47.98)	48.67 (40.43–56.95)	45.45 (42.95–47.98)

Data are presented as means (95% confidence interval)

*AUC* area under the curve, *PPV* positive predictive value, *NPV* negative predictive value

Finally, based on our model, we designed a corresponding user-friendly online calculator for clinicians to use in clinical practice and to facilitate independent external validation of our model (http://www.hnf1bproject.com). The result of calculation is a probability of having *HNF1B* mutation.

## Discussion

The diagnosis of *HNF1B*-associated disease is a challenging task due to high phenotypic variability, incomplete penetrance, frequent absence of family history, and possible overlap with other conditions [9]. Given that, an appropriate selection of those patients who likely have a high diagnostic yield for targeted genetic testing is crucial. At this moment, there is no accurate tool that can assist clinicians in the pregenetic screening of patients. In this study, we developed a predictive model for *HNF1B* mutations, and a corresponding calculator, which could be a reliable and efficient tool in the selection of pediatric patients with CAKUT for genetic testing.

The discriminative power of the model is good. Importantly, our model outperformed the existing *HNF1B* score (AUC 0.85 vs. 0.71). However, it must be remembered that Faguer’s score favors negative selection. The negative predictive value (NPV) in the development study for the French cohort was 99% [9], whereas in the validation study performed in a large UK dataset, it was 85% [10]. Notably, in our study, NPV of the Faguer’s score was 61% compared to 75% achieved by our model. As shown by Clissold et al. [10] and Lim et al. [11], the use of the proposed cutoff of ≥ 8 points in Faguer’s score may erroneously indicate the absence of mutations in children. Additionally, in the study by Raaijmakers et al. [12], the authors retrospectively applied this score in their cohort and found that it would have missed some patients carrying causative mutations. This might be due to the dynamic phenotype of the disease and the subsequent development of some phenotypic features.

Our predictive model, compared with the screening criteria of Faguer and Raaijmakers, is more applicable to routine clinical practice. The *HNF1B* score is very complex and requires much data, some of which are not available on a routine basis [12]. Raaijmakers’ criteria, however, have not been developed in the form of scoring, which restricts their use and validation. In this respect, our model and a corresponding online calculator are user friendly. It includes only five items that are easily available. The development of a calculator should make the model readily accessible and commonly used in practice at the bedside. In contrast to Faguer’s score, which is based on an arbitrarily developed scoring system and testing the optimal cutoff point, we intended to build a predictive model in which a decision is made by an algorithm. For this purpose, we applied efficient and advanced statistics for feature selection and model building.

Our results are not surprising since, like Faguer’s study [9], we showed that hypomagnesemia and pancreatic anomalies are relevant parameters. The selection of bilateral kidney anomalies in our model is also in concordance with the findings of Raaijmakers et al. [12], who considered this feature to be the most predictive in terms of kidney phenotype. In the same study, hypomagnesemia had a high relative risk for *HNF1B*-associated disease. Similar to our observation, Raaijmakers et al. suggest that *HNF1B* analysis should be considered in all patients with bilateral kidney anomalies and hypomagnesemia, irrespective of age at presentation, extrarenal symptoms, or the presence of an affected family member. In fact, bilateral defects are typical for *HNF1B* nephropathy [7, 8, 11, 12, 23–25]. In line with Raaijmakers et al. [12], a family history of kidney disease and/or DM was not discriminatory in our study.

Importantly, hypomagnesemia was found to be a highly selective parameter for *HNF1B* mutations, which is in concordance with other studies [9, 12]. In this study, we provide a proof of concept developed in an earlier study [16]. Here, we show that hypomagnesemia, according to age-appropriate norms, had better discriminatory value than the age-independent cutoff of 0.7 mmol/l. We also demonstrated that hypomagnesemia may be an early and common manifestation in individuals with *HNF1B* and, as such, could serve as a predictor at a young age. Adalat et al. [23] were the first to find an association between hypomagnesemia and *HNF1B* mutations. In a later study, the authors claimed that this abnormality develops with increasing age, and it is usually diagnosed at a later age [26]. Here, we confirm that the prevalence of hypomagnesemia increases with age, but noteworthy, we found hypomagnesemia in a substantial proportion of *HNF1B* patients in younger ages. The explanation for these conflicting results is the utilization of appropriate lower limits of sMg in our study instead of one cutoff value for sMg. Furthermore, we analyzed FEMg and proposed this parameter as a predictor of *HNF1B*. Considering the high selective value of hypomagnesemia, one could expect that hypermagnesuria may add to the prediction. It seems clear that impairment of tubular transport of magnesium should be assessed in pairs (i.e., sMg and FEMg), as it provides more information about magnesium handling [16]. For example, some patients may have hypermagnesuria with an intact sMg level, as the relationship between sMg concentration and FEMg is dynamic [27]. Therefore, magnesium wasting in the presence of normomagnesemia may indicate *HNF1B* nephropathy.

In this study, pancreatic anomalies were reported in 11.93% of pediatric patients and were selected as a valuable parameter. In this respect, our finding is quite unexpected. In a study of a large group of children, pancreatic anomalies were detected in only 0.9% of patients, while in adults, this feature was much more common (9.8%) [10]. Similarly, Raaijmakers et al. [12] reported this manifestation in only one patient, and this item did not receive any recognition. The inconsistency in reports could be partially explained by the fact that anatomical anomalies may be detected more commonly in adults, as abdominal computed tomography (CT) or magnetic resonance imaging is conducted more easily in this population. In a case series, pancreatic malformations were detected in three out of four patients, as CT was performed either to visualize kidney cysts or during an episode of liver derangement as part of differential diagnostics [28]. Currently, there are no data to advocate for CT scans in patients suspected of *HNF1B* to detect pancreatic anomalies. Therefore, the pancreas should be formally assessed by ultrasound in every patient with CAKUT. Additionally, in children with loose stools and/or failure to thrive, it is suggested that fecal elastase-1 be included in the work-up, which could serve as a screening marker of pancreas involvement [29].

There are conflicting results for DM, which in our study and in the study by Clissold et al. [10] were discriminative based on *OR*. In contrast, other authors [12, 25] did not find this parameter useful for selection. This discrepancy may possibly be due to the age of the studied population. Due to the higher prevalence of DM in adults, its selective power may be diminished. Importantly, maturity-onset diabetes in the young type 5 (MODY5), which is associated with *HNF1B* mutations, typically appears in adolescence and early adulthood; thus, this manifestation is usually absent when considering molecular testing in young children. Despite being discriminative, this parameter was not selected as an important feature and was not included in our model. On the other hand, this is not an unexpected finding, as the highest likelihood of *HNF1B* mutation identification in children with both MODY5 and kidney malformations is only 38% [30]. It is also no surprise that hyperuricemia was found to be irrelevant for prediction. In a previous study, we demonstrated its limited value due to a strong association of sUA with kidney function and PTH [17].

Not surprisingly, hypokalemia was absent in our cohort, yet serum potassium levels were associated with *HNF1B* mutations. Although this biochemical abnormality may not be frequently present in *HNF1B* nephropathy (3.5%), hypokalemia was also recognized by Faguer et al. [9]. As demonstrated by Adalat et al. [26], low potassium levels develop with age; thus, the chances of identifying it are higher at older ages. Given this, we shall not expect hypokalemia in young children. Despite this, potassium level was selected in our model, as it seems that discrete changes in the level of serum potassium may be informative.

We acknowledge some limitations of this study. For some patients, the data were not complete, and parameters were obtained/analyzed at unequal time points. As patients were recruited from different centers, the method of assessment of some laboratory parameters was nonuniform, and extrarenal abnormalities were not studied formally. Since the majority of patients had targeted *HNF1B* testing, we may not eliminate the possibility of the presence of other gene mutations responsible for polycystic kidney disease or CAKUT. The studied cohort had well-preserved kidney function, so our model may not be applicable to patients with more advanced CKD. We are also aware that this model has not been validated externally. The aim for future studies is the development of a model to predict *HNF1B* disease at a younger age, preferably for those < 2 years of age, when kidney anomalies may be the first and only manifestation of the disease, and the clinical utility would be the most appreciated. Unfortunately, the cohort size in this study was too small to generate a separate model for the young population of patients, which could be regarded as a limitation.

In conclusion, our clinical prediction tool for *HNF1B* mutations was developed in a large pediatric cohort using supervised machine learning methods. Based on the RF algorithm, this study established an accurate and simple predictive model that calculates an individual’s probability of having *HNF1B* mutation. Our tool would facilitate the selection of children and young adults with CAKUT with normal or mildly compromised kidney function for genetic testing, thereby improving the detection rate and reducing the costs of testing. We also showed that abnormalities in magnesium handling are important features of *HNF1B*-associated disease. Hence, the utilization of age-appropriate lower limits of sMg is highly recommended to achieve good model performance. Further work is needed to validate the model in a prospective study.

### Supplementary Information

Below is the link to the electronic supplementary material.

Graphical abstract (PPTX 295 KB)

Supplementary file2 (DOCX 175 KB)

Supplementary file3 (XLSX 42 KB)

## Data Availability

The dataset generated during and/or analyzed during the current study are available from the corresponding author on reasonable request.

## References

[1] Weber S, Moriniere V, Knüppel T, Charbit M, Dusek J, Ghiggeri GM, Jankauskiené A, Mir S, Montini G, Peco-Antic A, Wühl E, Zurowska AM, Mehls O, Antignac C, Schaefer F, Salomon R (2006). Prevalence of mutations in renal developmental genes in children with renal hypodysplasia: results of the ESCAPE study. J Am Soc Nephrol.

[2] Thomas R, Sanna-Cherchi S, Warady BA, Furth SL, Kaskel FJ, Gharavi AG (2011). HNF1B and PAX2 mutations are a common cause of renal hypodysplasia in the CKiD cohort. Pediatr Nephrol.

[3] Sanna-Cherchi S, Kiryluk K, Burgess KE, Bodria M, Sampson MG, Hadley D, Nees SN, Verbitsky M, Perry BJ, Sterken R, Lozanovski VJ, Materna-Kiryluk A, Barlassina C, Kini A, Corbani V, Carrea A, Somenzi D, Murtas C, Ristoska-Bojkovska N, Izzi C, Bianco B, Zaniew M, Flogelova H, Weng PL, Kacak N, Giberti S, Gigante M, Arapovic A, Drnasin K, Caridi G, Curioni S, Allegri F, Ammenti A, Ferretti S, Goj V, Bernardo L, Jobanputra V, Chung WK, Lifton RP, Sanders S, State M, Clark LN, Saraga M, Padmanabhan S, Dominiczak AF, Foroud T, Gesualdo L, Gucev Z, Allegri L, Latos-Bielenska A, Cusi D, Scolari F, Tasic V, Hakonarson H, Ghiggeri GM, Gharavi AG (2012). Copy-number disorders are a common cause of congenital kidney malformations. Am J Hum Genet.

[4] Vivante A, Kohl S, Hwang D-Y, Dworschak GC, Hildebrandt F (2014). Single-gene causes of congenital anomalies of the kidney and urinary tract (CAKUT) in humans. Pediatr Nephrol.

[5] Wühl E, van Stralen KJ, Verrina E, Bjerre A, Wanner C, Heaf JG, Zurriaga O, Hoitsma A, Niaudet P, Palsson R, Ravani P, Jager KJ, Schaefer F (2013). Timing and outcome of renal replacement therapy in patients with congenital malformations of the kidney and urinary tract. Clin J Am Soc Nephrol.

[6] Bockenhauer D, Jaureguiberry G (2016). HNF1B-associated clinical phenotypes: the kidney and beyond. Pediatr Nephrol.

[7] Decramer S, Parant O, Beaufils S, Clauin S, Guillou C, Kessler S, Aziza J, Bandin F, Schanstra JP, Bellanné-Chantelot C (2007). Anomalies of the TCF2 gene are the main cause of fetal bilateral hyperechogenic kidneys. J Am Soc Nephrol.

[8] Heidet L, Decramer S, Pawtowski A, Morinière V, Bandin F, Knebelmann B, Lebre A-S, Faguer S, Guigonis V, Antignac C, Salomon R (2010). Spectrum of HNF1B mutations in a large cohort of patients who harbor renal diseases. Clin J Am Soc Nephrol.

[9] Faguer S, Chassaing N, Bandin F, Prouheze C, Garnier A, Casemayou A, Huart A, Schanstra JP, Calvas P, Decramer S, Chauveau D (2014). The HNF1B score is a simple tool to select patients for HNF1B gene analysis. Kidney Int.

[10] Clissold R, Shields B, Ellard S, Hattersley A, Bingham C (2015). Assessment of the HNF1B score as a tool to select patients for HNF1B genetic testing. Nephron.

[11] Lim SH, Kim JH, Han KH, Ahn YH, Kang HG, Ha I-S, Il CH (2020). Genotype and phenotype analyses in pediatric patients with HNF1B mutations. J Clin Med.

[12] Raaijmakers A, Corveleyn A, Devriendt K, van Tienoven TP, Allegaert K, Van Dyck M, van den Heuvel L, Kuypers D, Claes K, Mekahli D, Levtchenko E (2015). Criteria for HNF1B analysis in patients with congenital abnormalities of kidney and urinary tract. Nephrol Dial Transplant.

[13] Ridefelt P, Aldrimer M, Rödöö P-O, Niklasson F, Jansson L, Gustafsson J, Hellberg D (2012). Population-based pediatric reference intervals for general clinical chemistry analytes on the Abbott Architect ci8200 instrument. Clin Chem Lab Med.

[14] Colantonio DA, Kyriakopoulou L, Chan MK, Daly CH, Brinc D, Venner AA, Pasic MD, Armbruster D, Adeli K (2012). Closing the gaps in pediatric laboratory reference intervals: a CALIPER database of 40 biochemical markers in a healthy and multiethnic population of children. Clin Chem.

[15] Ridefelt P, Hilsted L, Juul A, Hellberg D, Rustad P (2018). Pediatric reference intervals for general clinical chemistry components - merging of studies from Denmark and Sweden. Scand J Clin Lab Invest.

[16] Kołbuc M, Leßmeier L, Salamon-Słowińska D, Małecka I, Pawlaczyk K, Walkowiak J, Wysocki J, Beck BB, Zaniew M (2020). Hypomagnesemia is underestimated in children with HNF1B mutations. Pediatr Nephrol.

[17] Kołbuc M, Bieniaś B, Habbig S, Kołek MF, Szczepańska M, Kiliś-Pstrusińska K, Wasilewska A, Adamczyk P, Motyka R, Tkaczyk M, Sikora P, Beck BB, Zaniew M (2021). Hyperuricemia is an early and relatively common feature in children with HNF1B nephropathy but its utility as a predictor of the disease is limited. J Clin Med.

[18] Seeman T, Fořtová M, Sopko B, Průša R, Pohl M, John U (2019). Hypomagnesaemia is absent in children with autosomal dominant polycystic kidney disease. Ann Clin Biochem.

[19] Schwartz GJ, Muñoz A, Schneider MF, Mak RH, Kaskel F, Warady BA, Furth SL (2009). New equations to estimate GFR in children with CKD. J Am Soc Nephrol.

[20] Schwartz GJ, Brion LP, Spitzer A (1987). The use of plasma creatinine concentration for estimating glomerular filtration rate in infants, children, and adolescents. Pediatr Clin North Am.

[21] Levey AS, Stevens LA, Schmid CH, Zhang YL, Castro AF, Feldman HI, Kusek JW, Eggers P, Van Lente F, Greene T, Coresh J (2009). A new equation to estimate glomerular filtration rate. Ann Intern Med.

[22] National Kidney Foundation (2002). K/DOQI clinical practice guidelines for chronic kidney disease: evaluation, classification, and stratification. Am J Kidney Dis.

[23] Adalat S, Woolf AS, Johnstone KA, Wirsing A, Harries LW, Long DA, Hennekam RC, Ledermann SE, Rees L, van’t Hoff W, Marks SD, Trompeter RS, Tullus K, Winyard PJ, Cansick J, Mushtaq I, Dhillon HK, Bingham C, Edghill EL, Shroff R, Stanescu H, Ryffel GU, Ellard S, Bockenhauer D (2009). HNF1B mutations associate with hypomagnesemia and renal magnesium wasting. J Am Soc Nephrol.

[24] Okorn C, Goertz A, Vester U, Beck BB, Bergmann C, Habbig S, König J, Konrad M, Müller D, Oh J, Ortiz-Brüchle N, Patzer L, Schild R, Seeman T, Staude H, Thumfart J, Tönshoff B, Walden U, Weber L, Zaniew M, Zappel H, Hoyer PF, Weber S (2019). HNF1B nephropathy has a slow-progressive phenotype in childhood-with the exception of very early onset cases: results of the German Multicenter HNF1B Childhood Registry. Pediatr Nephrol.

[25] Madariaga L, García-Castaño A, Ariceta G, Martínez-Salazar R, Aguayo A, Castaño L (2019). Variable phenotype in HNF1B mutations: extrarenal manifestations distinguish affected individuals from the population with congenital anomalies of the kidney and urinary tract. Clin Kidney J.

[26] Adalat S, Hayes WN, Bryant WA, Booth J, Woolf AS, Kleta R, Subtil S, Clissold R, Colclough K, Ellard S, Bockenhauer D (2019). HNF1B mutations are associated with a Gitelman-like tubulopathy that develops during childhood. Kidney Int Rep.

[27] Viering DHHM, de Baaij JHF, Walsh SB, Kleta R, Bockenhauer D (2017). Genetic causes of hypomagnesemia, a clinical overview. Pediatr Nephrol.

[28] Motyka R, Kołbuc M, Wierzchołowski W, Beck BB, Towpik IE, Zaniew M (2020). Four Cases of Maturity Onset Diabetes of the Young (MODY) Type 5 associated with mutations in the hepatocyte nuclear factor 1 beta (HNF1B) gene presenting in a 13-year-old boy and in adult men aged 33, 34, and 35 years in Poland. Am J Case Rep.

[29] Clissold RL, Fulford J, Hudson M, Shields BM, McDonald TJ, Ellard S, Hattersley AT, Bingham C (2018). Exocrine pancreatic dysfunction is common in hepatocyte nuclear factor 1β-associated renal disease and can be symptomatic. Clin Kidney J.

[30] Sztromwasser P, Michalak A, Małachowska B, Młudzik P, Antosik K, Hogendorf A, Zmysłowska A, Borowiec M, Młynarski W, Fendler W (2020). A cross-sectional study of patients referred for HNF1B-MODY genetic testing due to cystic kidneys and diabetes. Pediatr Diabetes.

