# Supplementary material for: Development of a tool for predicting HNF1B mutations in children and young adults with congenital anomalies of the kidneys and urinary tract
Source: Pediatr Nephrol. 2024 Jan 10;39(6):1847–58. doi: 10.1007/s00467-023-06262-9 (PMC11026189; doi:10.1007/s00467-023-06262-9)
Supplement: Supplementary file 1 — Graphical abstract (PPTX 295 KB) [file 467_2023_6262_MOESM1_ESM.pptx]

## Slide 1
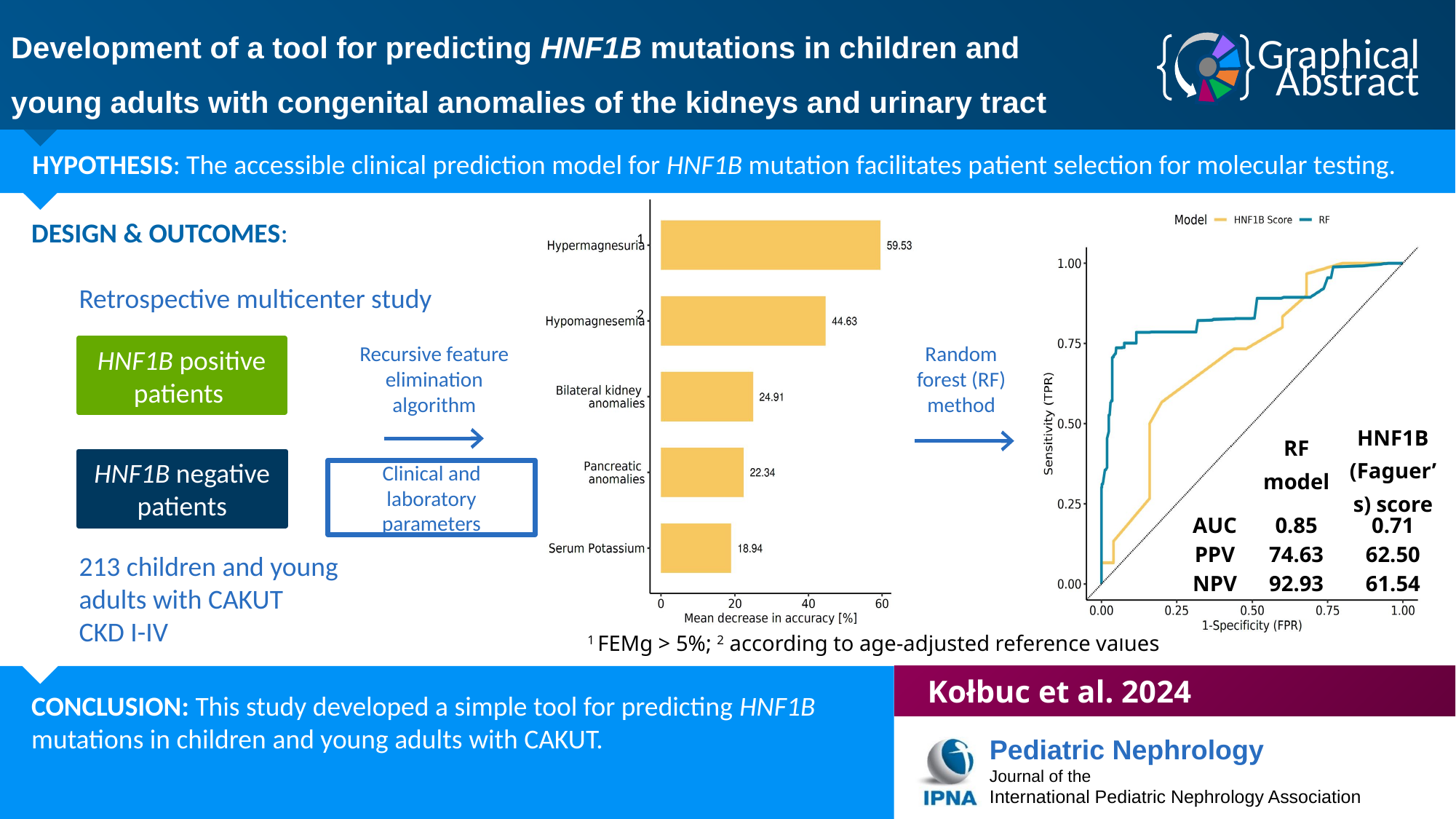

Development of a tool for predicting HNF1B mutations in children and young adults with congenital anomalies of the kidneys and urinary tract
HYPOTHESIS: The accessible clinical prediction model for HNF1B mutation facilitates patient selection for molecular testing.
DESIGN & OUTCOMES:
1
Retrospective multicenter study
2
Recursive feature elimination algorithm
Random forest (RF) method
HNF1B positive patients
| | RF model | HNF1B (Faguer’s) score |
| --- | --- | --- |
| AUC | 0.85 | 0.71 |
| PPV | 74.63 | 62.50 |
| NPV | 92.93 | 61.54 |
HNF1B negative patients
Clinical and laboratory parameters
213 children and young adults with CAKUT
CKD I-IV
1 FEMg > 5%; 2 according to age-adjusted reference values
Kołbuc et al. 2024
CONCLUSION: This study developed a simple tool for predicting HNF1B mutations in children and young adults with CAKUT.
