# Supplementary material for: Development of a tool for predicting HNF1B mutations in children and young adults with congenital anomalies of the kidneys and urinary tract
Source: Pediatr Nephrol. 2024 Jan 10;39(6):1847–58. doi: 10.1007/s00467-023-06262-9 (PMC11026189; doi:10.1007/s00467-023-06262-9)
Supplement: Supplementary file 2 — Supplementary file2 (DOCX 175 KB) [file 467_2023_6262_MOESM2_ESM.docx]

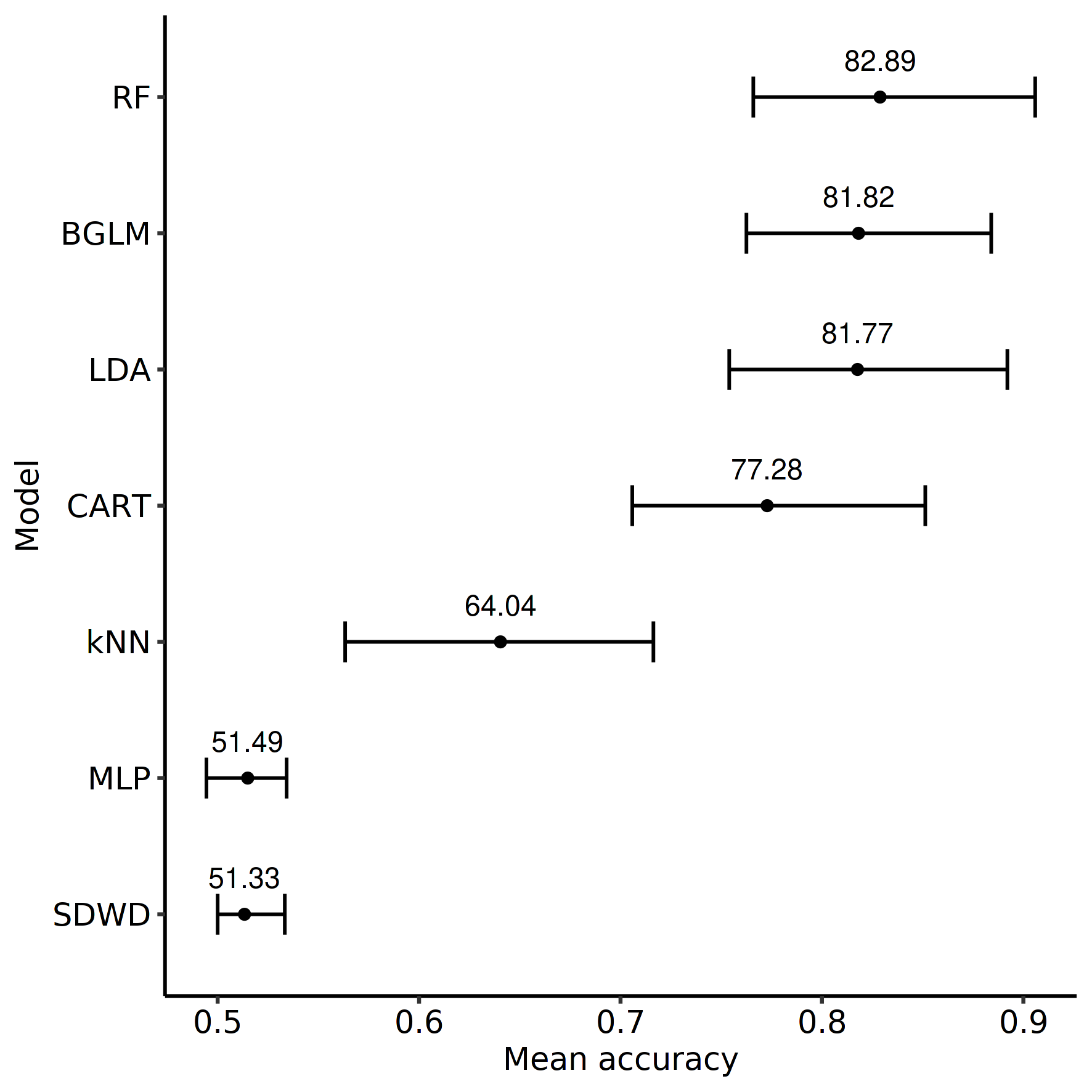


**Supplementary Fig. S1:** Performance of tested algorithms. Data presented as mean accuracy, 1st and 3rd quartile

random forest, RF; k-nearest neighbors, k-NN; support vector machine, SVM; classification and regression trees, CART; Bayes generalized linear model, BGLM; linear discriminant analysis, LDA; multilayer perceptron, MLP; sparse distance weighted discrimination, SDWD; shrinkage discriminant analysis, SDA


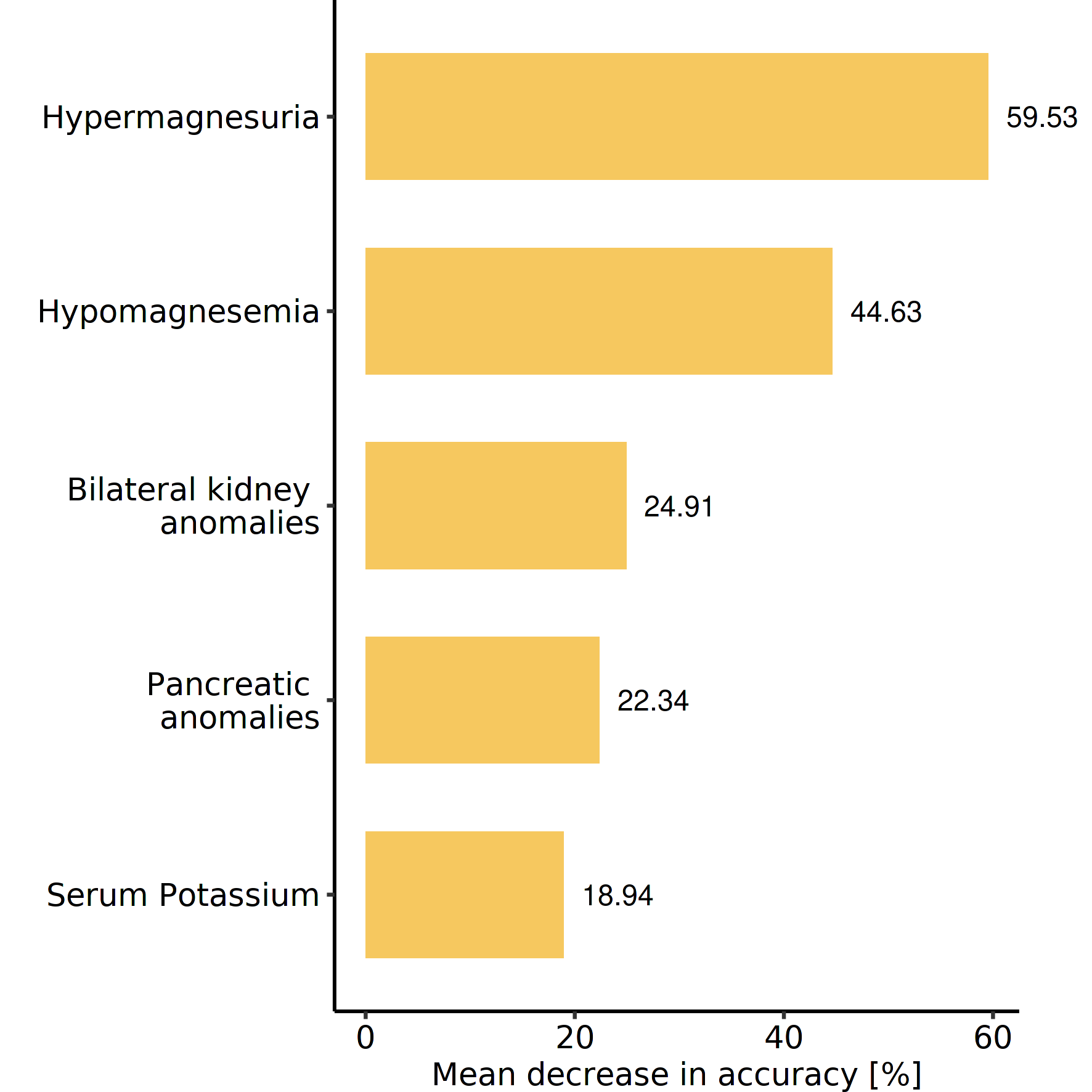


**Supplementary Fig. S2** Importance of the variables, defined by mean decrease in accuracy, selected for the predictive model
